# Psychiatric Illness and Clinical Negligence: When Can “Secondary Victims” Successfully Claim for Damages? Recent Developments from the United Kingdom

**DOI:** 10.1007/s11673-024-10346-y

**Published:** 2024-05-22

**Authors:** Edward S. Dove

**Affiliations:** School of Law, University of Edinburgh, Old College, South Bridge, Edinburgh, EH8 9YL UK

**Keywords:** Accident, Clinical negligence, Duty of care, Negligence, Secondary victims, Tort

## Abstract

On January 11, 2024, the United Kingdom (U.K.) Supreme Court rendered its judgment in *Paul v Royal Wolverhampton NHS Trust*, restricting the circumstances in which “secondary victims” can successfully claim for damages in clinical negligence cases. This ruling has provided welcome clarity regarding the scope of negligently caused “pure” psychiatric illness claims, but the judgment may well prove controversial. In this article, I trace the facts and opinion from the majority and also discuss an important dissenting opinion. I then reflect on what the ruling means for psychiatric illness claims by secondary victims, and more broadly on the implications for clinical negligence law. I suggest that while much-needed clarity has been injected in this area of the law, it is difficult, reading the majority of the Supreme Court’s emphasis on the restricted scope of a medical practitioner’s duty, to envision a scenario in which secondary victim could ever succeed in a clinical negligence context.

## Introduction

Much welcome and needed clarity has been provided in the United Kingdom (U.K.) for personal injury practitioners, clinical negligence lawyers, as well as the medical profession regarding the scope of negligently caused “pure” psychiatric illness claims. Coupled with this clarity, however, is some concern about the scope of the law providing justice for claimants whom the law considers to be “secondary victims.”

On January 11, 2024, the U.K. Supreme Court rendered its judgment in *Paul v Royal Wolverhampton NHS Trust* ([2024] UKSC 1) (“*Paul*”),^1^ holding 6-1 (with Lord Burrows dissenting) that the appeal of the Claimants (which were three conjoined cases and appeals, and which I shorten to *Paul*, *Polmear*, and *Purchase*) should be dismissed, and restricting the circumstances in which a person, who witnesses the death of or serious injury to a loved one as a result of an illness that proper medical treatment would have prevented, can bring a claim as a secondary victim. The three cases each involved a claim from a deceased’s close relative (what is termed a “secondary victim”) for psychiatric injury caused by witnessing, or attending shortly after, the death of a loved one (what is termed the “primary victim”), where the death was allegedly caused by the defendant’s earlier clinical negligence.

The critical question on which the validity of the claims depended was whether a medical practitioner, in providing medical services to a patient, not only owes a duty to the patient to take care to protect the patient from harm *but also* owes a duty to close members of the patient’s family to take care to protect them against the risk of injury that they might suffer from the experience of witnessing the death or injury of their relative from an illness caused by the doctor’s negligence (*Paul*, [22]).

To answer this critical question, the Court was asked to recognize, as analogous to accident cases regarding the recovery of damages by secondary victims, a category of cases in which a recognized (or recognizable) psychiatric illness is sustained by a secondary victim as a result of witnessing a death or manifestation of injury which is not caused by an external, traumatic event in the nature of an accident but rather is the result of a pre-existing injury or disease. As I discuss below, the majority of the Court did not consider that such cases are analogous and thus, ruled that there does not exist the proximity in the relationship between the parties necessary to give rise to a duty of care.

In what follows, I trace the facts and finding of the majority of the Court, in an opinion jointly delivered by Lord Leggatt and Lady Rose (with whom Lord Briggs, Lord Sales, and Lord Richards agreed). I then reflect on what the ruling means for psychiatric illness claims by secondary victims both within the U.K. and beyond and more broadly on the implications for clinical negligence law. I suggest that while much-needed clarity has been injected in this area of the law, it is difficult, reading the majority of the Supreme Court’s emphasis on the restricted scope of a medical practitioner’s duty, to envision a scenario in which secondary victim could ever succeed in a clinical negligence context.

## The Case

### Factual Background

The facts of the three conjoined cases, briefly stated, are as follows.^2^

In *Paul*, Mr Parminder Singh Paul collapsed and died from a cardiac arrest on January 26, 2014 while shopping with his daughters (aged nine and twelve). His daughters suffered psychiatric injury as a result of witnessing their father’s collapse (he fell backwards and hit his head on the pavement), its upsetting aftermath (they watched paramedics performing chest compressions on him, and putting a foil blanket over him), and his death. It was subsequently alleged that his death had occurred as a result of a negligent failure by the Royal Wolverhampton NHS Trust to diagnose and treat his significant coronary artery disease fourteen months earlier.

In *Polmear*, Esmee Polmear, aged seven, collapsed and died on July 1, 2015 during a school trip. Esmee’s collapse, unsuccessful attempts at resuscitation (including by her father), and her death, were witnessed by her mother and father, both of whom suffered post-traumatic stress disorder (“PTSD”) and major depression as a result. Esmee’s cause of death was pulmonary veno-occlusive disease. She had been seen by a consultant paediatrician on December 1, 2014 due to episodes in which she could not breathe, appeared pale, and turned blue. In January 2015, some tests were carried out and the paediatrician wrongly concluded that the symptoms were likely related to exertion only. The Royal Cornwall Hospitals Trust admitted that there was a failure to diagnose the condition in mid-January 2015.

In *Purchase*, Evelyn Purchase, aged twenty, died on April 7, 2013 from extensive bilateral pneumonia with pulmonary abscesses. Evelyn was found by her mother at home motionless on her bed with the house telephone in her hand, staring at the ceiling and not moving. Her mother’s attempts at cardiopulmonary resuscitation (“CPR”) failed; when she opened Evelyn’s mouth to attempt mouth-to-mouth resuscitation, blood and bodily fluids spilled out of Evelyn’s mouth and nose. It was determined that she had died about five minutes before being found. Evelyn’s mother subsequently discovered Evelyn had tried calling her on her mobile phone; the voice message was the sound of Evelyn’s dying breaths, which continued for four minutes and thirty-seven seconds. Her mother developed PTSD, severe chronic anxiety, and depression. It was alleged that Evelyn had presented to an out-of-hours clinic with symptoms of severe pneumonia on April 4, 2013 (having been unwell for several weeks and having made two previous visits to her GP) and that there was a negligent failure by the treating doctor to assess and treat her, as a result of which she died.

Thus, it was an agreed fact that each of the claimants was suffering from a medically recognized psychiatric illness, and in each case, there was clinical negligence in failing to diagnose and treat the primary victim’s life-threatening condition, which led, some time after the breach of the duty, to the unexpected death of the primary victim. Each secondary victim was a close relative who either witnessed the death or came upon the primary victim immediately after their death.

### Legal Background

Personal injury practitioners and clinical negligence lawyers have long lamented that the law in the U.K. relating to secondary victim claims is complex and inconsistent. Some aspects have been clear for a long time, though. For instance, the general rule is that the death or severe injury of a person cannot be complained of as an injury by another person (*qua* secondary victim).^3^ However, under the Fatal Accidents Act 1976, as amended, certain dependants of a person whose death is caused by a wrongful act, neglect, or default the right to sue and recover damages from the person who (if death had not ensued) would have been liable to the deceased. These damages cover financial loss. The deceased’s spouse or partner, or parents if the child was an unmarried minor, can also claim damages for bereavement but these are limited to a fixed sum. The Act does not provide a remedy for physical or psychological harm caused to relatives or others by the death.

On the other side, a line of cases has indicated that damages may be recovered for personal injury (often psychiatric illness) arising from the death or injury of another person where the claimant has witnessed the wrongful death or injury (or threat of such death or injury) to someone they love. The *scope* of the category of these cases was at the heart of these three appeals. As noted above, the legal question for the Supreme Court was: ought the law extend its (incremental) reach to allow claimants to recover damages for personal injury caused by witnessing the death or injury of a close relative, not in an accident but from a medical condition which the defendant negligently failed to diagnose and treat? The question seemingly required the Court to balance the right of a secondary victim to bring a claim against the need to limit recoverability for secondary victims; otherwise, defendants (often medical practitioners’ employer) would be subject to multiple civil claims arising from one negligent act or omission.

As the Court noted, the extant leading authority for legal proximity in the U.K. is *Alcock v Chief Constable of the South Yorkshire Police* ([1992] 1 AC 310) (“*Alcock*”). This case involved a claim from a group of people who had witnessed the Hillsborough disaster^4^ (some in person, some watching events play out on television). In his judgment, Lord Oliver set out five rules of recoverability which have become to be known as the five “control mechanisms,” which a secondary victim must prove in order to recover damages. These are:

1. There must be a close tie of love and affection between the primary and secondary victim (this is usually a marital or parental relationship);
2. The psychiatric injury must arise from a sudden and unexpected shock to the secondary victim’s nervous system [or phrased more accurately and in modern parlance as a recognized or recognizable psychiatric illness] (i.e. as a result of a shocking event rather than gradual realization over the course of time);
3. The secondary victim must be personally present at the scene or immediate aftermath (physical proximity);
4. The injury to the secondary victim must arise from the death, extreme danger to, or injury of the primary victim; and
5. Lastly, there must be not only an element of physical proximity to the event but a close temporal connection between the accident/event and the secondary victim’s perception of it combined with a close relationship of affection between the claimant and the primary victim (this does not mean there must be a close temporal connection between the defendant’s negligence and the accident/event which causes the claimant psychiatric injury).

It is the fifth “control mechanism” that was the focus of the judgment in *Paul*.

Two other cases from the House of Lords have set the requirements under the common law of England and Wales for a successful claim by someone who suffers from psychiatric illness in connection with the death or injury of another person. In *McLoughlin v O’Brian* ([1983] 1 AC 410) (“*McLoughlin*”), the claimant was not present at the scene of a road accident but saw injuries caused to members of her family shortly afterwards at the hospital. She suffered from both physical and psychological illnesses, and the House of Lords ruled unanimously that she was entitled to damages, although Lord Wilberforce acknowledged that allowing the claim was “upon the margin of what the process of logical progression would allow” (*McLoughlin*, 419G) and identified several elements inherent in any claim that had to be considered to keep the liability of the defendant within reasonable bounds.

In *Frost v Chief Constable of South Yorkshire* ([1999] 2 AC 455) (“*Frost*”), another Hillsborough disaster case, the House of Lords considered whether police officers who were present at Hillsborough stadium who suffered psychiatric illness could recover compensation. The House of Lords answered in the negative: none of the claimants had a close tie of love and affection with any of those killed or physically injured, as set out in the *Alcock* control mechanisms. Moreover, they held that the category of primary victims is limited to persons exposed (or who perceive themselves to be exposed) to *physical* danger and thus could not encompass the claimants.

In the years since *Alcock*, English courts have sought to interpret Lord Oliver’s “control mechanisms” and apply them to different circumstances; many of the cases involved accidents (mostly road traffic accidents). Of most significance to these appeals is the Court of Appeal decision in *Taylor v A Novo (UK) Ltd* ([2013] EWCA Civ 194) (“*Novo*”), which was an accident case. In that case, the claimant’s mother was injured at work when a stack of racking boards fell on top of her. Her employer admitted negligence. Three weeks later, after seemingly recovering well, she collapsed and died (caused by a pulmonary embolism resulting from a deep vein thrombosis due to the injuries sustained in the accident) in the presence of her daughter. The daughter suffered PTSD as a result of witnessing her mother’s death. She brought a claim as a secondary victim. The Court of Appeal found in favour of the defendant. Lord Dyson held that proximity was lacking because the claimant was not present at the scene of the accident (when the racking boards fell on her mother) and was not involved in its immediate aftermath. As such, the necessary element of temporal proximity – the fifth “control mechanism”—was lacking. In other words, the relevant event was the accident alone and one could not treat the later death, which was witnessed by the secondary victim, as the event for these purposes. There was concern that allowing the claim would mean claimants could cover damages even if the primary victim’s death occurred months or possibly years after the accident; there was also concern that allowing recovery would extend the scope of liability to secondary victims considerably further than in previous cases and policy reasons articulated by the House of Lords in *Frost* militated against any such extension. In each of *Paul*, *Polmear*, and *Purchase*, the Court of Appeal held itself bound by the decision in *Novo* to find for the defendants and to deny liability.

In their judgment, Lord Leggatt and Lady Rose also considered a number of cases in the thirty years since *Alcock* that involved claims made by secondary victims in clinical negligence cases, although these cases rarely involved discussion of whether damages could in principle be recovered in the absence of an accident caused by the defendant’s negligence and whether in principle the rules developed in accident cases ought to be applied.^5^

### The U.K. Supreme Court’s Ruling

The majority made clear that the previous line of cases developed in relation to accidents could not apply to clinical negligence cases without considering the general principles that determine when a medical practitioner owes a duty of care to someone other than their patient. Equally, the Court was concerned with defining the limits on the recovery of damages by secondary victims “to avoid distinctions which would offend most people’s sense of justice” (*Paul*, [49]).

The majority distinguished the line of cases discussed from clinical negligence cases like those under the appeal. As they put it: “In these cases, the event (or its aftermath) witnessed by the secondary victim is generally not an accident; it is the suffering or death of their relative from illness,” or what they termed in shorthand as a “medical crisis” (*Paul*, [53]). In their view, witnessing a negligently caused medical crisis (or its aftermath) cannot in principle found a claim for damages by a secondary victim; rather, such a claim can only lie *where the triggering (i.e. relevant) event is an accident* in the sense that it is an “unexpected and unintended event which cause[s] injury (or risk of injury) by violent external means to one or more primary victims” (*Paul*, [52]). The line of “accident cases” could not be interpreted as covering or otherwise extending to negligently caused medical crises. Beyond the “accident” requirement, the majority held that for a person to qualify as a secondary victim where that person has sustained a recognized (or recognizable) psychiatric injury or illness that was reasonably foreseeable,^6^ there must be a sufficiently close tie of love and affection between the secondary victim and the person or persons suffering physical injury, and the psychiatric injury suffered must have been caused by their direct perception (i.e. with their own senses, in-person) of the “accident” or its immediate aftermath.

This reasoning meant that the majority overruled the “first manifestation of the damage” test, which is that the relevant event for the purposes of a claim by a secondary victim must be the first manifestation of damage to the primary victim. Though it was compatible with *Novo*, it was inconsistent with the reasoning in that case. The majority ruled that a secondary victim needs to witness an accident or its immediate aftermath for recovery to be possible for psychiatric injury. Thus, the Court held that a previous clinical negligence case (*Walters*^5^) was wrongly decided because the brain damage and death of Mrs Walter’s baby were not caused by an accident, and that the previous clinical negligence cases of *Sion*, *Shorter*, and *Ronayne*,^5^ although correctly decided, were decided on the wrong basis and that they should all have been dismissed because the claimants did not witness an accident.

The majority identified several crucial contrasts with accident cases (see [111]-[117] of the judgment). First, in many (but not all) medical crisis cases, there is no discrete event comparable to accident; the length of time for which symptoms of injury or disease last before a person recovers or dies is entirely variable and can range from minutes to weeks. This causes uncertainty about what qualifies as an “event” capable of founding a claim in negligence. Second, the extent to which the experience of witnessing the injury or illness of a close member is traumatic is entirely variable. Third, in cases where the claimant was not present at the scene of any accident, any psychiatric injury which the claimant suffers can only be of a secondary nature caused by witnessing the injury, illness, or death of another person, and “[a]llowing the claimant to recover compensation cannot therefore be justified by the practical impossibility and injustice of otherwise having to distinguish between injury caused by fear for the claimant’s own safety and injury caused by fear for the safety of a close family member” (*Paul*, [114]). Fourth, extending the scope of allowable claims by secondary victims to situations where the claimant witnesses the death or illness of a relative from disease would give rise to unacceptable and unfair differences in treatment between different categories of claimant. Fifth and finally, the majority expressed reservation that hospitals would be exposed to legal liability in end-of-life care scenarios if there were risk that, if it is said that the death ought to have been prevented, they permitted family members seeing and remaining with the patient.

How, then, ought “secondary victims” claim for damages in the context of a medical crisis and where the defendant is a medical practitioner? The majority held that it must be considered whether a duty of care is owed “by reference to the general principles applicable to this type of case” (*Paul*, [128]). As applied to the medical context, this means that there must not only exist reasonable foreseeability of harm; there must also exist a necessary “proximity” in the relationship between the claimant and defendant medical practitioner to make it just to impose such a duty. The scope of the duty of care will vary with the circumstances and will depend on the purpose for which the service is provided to the patient.

While recognizing that there are some circumstances in which the duty of care owed by a medical practitioner may extend beyond the health of their patient to include other people (e.g. when the patient has an infectious disease) (*Paul*, [134]), the majority considered it a step too far to recognize as a general principle that a medical practitioner owes a duty of care to members of the patient’s close family to take care to protect them against the risk of illness from the experience of witnessing the medical crisis of their relative arising from that practitioner’s negligence:

As regards other factors relevant to whether the necessary relationship of proximity exists, the extent of the control which a doctor may be seen as having over the risk of injury to members of the patient’s family and the directness of the causal link between the doctor’s negligence and the materialisation of that risk will depend upon the particular facts of the case (*Paul*, [137]).

But these factors are certainly suggested to be construed narrowly:

We are not able to accept that the responsibilities of a medical practitioner, and the purposes for which care is provided, extend to protecting members of the patient’s close family from exposure to the traumatic experience of witnessing the death or manifestation of disease or injury in their relative. To impose such a responsibility on hospitals and doctors would go beyond what, in the current state of our society, is reasonably regarded as the nature and scope of their role (*Paul*, [138]).

It is worth briefly touching on the sole dissenting opinion from Lord Burrows, who was the Law Commissioner in charge of a report twenty-five years prior entitled *Liability for Psychiatric Illness* (Law Commission of England and Wales 1998). In his opinion, expressed over a lengthy 107 paragraphs, there were several reasons why the appeals ought to have been allowed and why *Novo* ought to be considered incorrectly decided. Most importantly, in his view, the relevant (triggering) event should be viewed as the death of the primary victim (and thus he also rejected the relevant event as the “first manifestation of the damage”), and applying existing proximity and control factors, which were satisfied for all the claimants, it followed that a relevant duty of care was owed to the relatives in all three cases. On Lord Burrows’ approach, then, there is no requirement for there to be an “accident”: the exception to the general rule can be extended to cases involving clinical negligence as an appropriate incremental development of the common law in this area.

It is also interesting to note that Lord Carloway, Lord President of the Court of Session and Lord Justice General in Scotland, provided a separate concurring opinion (with whom Lord Sales agreed) observing the difference in Scots law in relation to the right to claim damages caused by the death of another person but also observing that had Scots law been applied in these appeals, “the same result in relation to the present claimants would have been reached” (*Paul*, [253]).

## Discussion

The majority decision in *Paul* seems to align with other recent decisions by the Supreme Court that put stricter boundaries around the scope of the medical profession’s and healthcare system’s liability to both patients and third parties. If *Montgomery v Lanarkshire Health Board* ([2015] UKSC 11) is seen as the highwater mark of valorizing patients’ rights, with an enhanced requirement of disclosure of risks by medical practitioners to satisfy the criteria of an informed consent in medical treatment, judgments from the past few years signal that the pendulum has begun to swing the other way. The first signal was in *Meadows v Khan* ([2021] UKSC 21), where the Supreme Court held that there must be a sufficient nexus between the claimant’s harm claimed for and the defendant’s duty of care. Applied to that case, it meant that the doctor was liable only for losses within the scope of her duty of care to advise the claimant about being a carrier of the haemophilia gene; there was no liability for costs associated with the diagnosis of her child’s autism. The second signal was in *McCulloch v Forth Valley Health Board* ([2023] UKSC 26), where the Supreme Court held that whether a treatment is a reasonable alternative is to be determined by application of the professional practice test and that a doctor is not obliged to tell a patient about treatments that the doctor does not consider reasonable (applying the professional practice test), even where the doctor is aware of an alternative body of opinion which considers the treatment to be reasonable. *Paul* may be the third signal of the swinging pendulum. As healthcare becomes increasingly complex and the expectations (and demands) of patients to have their complex conditions and comorbidities treated rise, and as the U.K.’s public healthcare systems come under increasing resource constraints, the judiciary may be playing the role of public resource liability guardian. As the majority put it, *Paul* raises a “fundamental question about the nature of the doctor’s role and the purposes for which medical care is provided to a patient” (*Paul*, [138]).

The ruling in *Paul* cannot be said to align with the law in other jurisdictions. Indeed, the majority took note that “there are significant differences between English, Australian, Canadian and New Zealand law, not to mention the laws of different states of the United States, concerning the recovery of damages for psychiatric harm suffered in connection with the death, injury or imperilment of another person caused by the defendant’s negligence,” and in consequence, made it “difficult and perhaps dangerous to draw any direct analogy” (*Paul*, [118]). Moreover, there was no Commonwealth authority cited to the Court which addressed the recoverability of such damages in cases of clinical negligence. In his dissent, Lord Burrows also noted that there are “significant differences” between the common law jurisdictions and that “it is therefore difficult and potentially misleading in this area to seek to draw lessons from the legal position in other common law jurisdictions” (*Paul*, [246]).

As ever, then, tort law gives rise to a wide range of approaches across jurisdictions. But I venture to say that *Paul*’s holding that a claimant cannot recover damages for personal injury as a secondary victim, unless the claimant witnessed an “accident” (or its immediate aftermath) caused by the defendant’s negligence, will be treated with some hesitation. The Supreme Court has helpfully simplified secondary victim claims by focusing analysis on the relevant event (in the majority’s view, this being an accident); whether its perception by the claimant caused psychiatric injury; and whether the psychiatric injury was reasonably foreseeable. However, while the Court was right “to avoid distinctions which would offend most people’s sense of justice” (*Paul*, [49]), it difficult to see how the distinction crafted between an “accident” and a “medical crisis” would not offend many or most people’s sense of justice. I am inclined to read the majority opinion as reflecting a dislike of the exception to the general principle that remedies ought not to be awarded to third parties for the effects of injuries to other people and as such, an effort to firmly limit the exception to “accident cases” under the *Alcock* control mechanisms.

Indeed, it is interesting to note that the majority acknowledged that counsel from the claimants had asked whether the rules governing claims by secondary victims arising from accidents could *ever* apply in a medical setting. But they ducked answering this, stating only that possible examples (e.g. a doctor injecting a patient with a wrong dose, inducing an acute adverse reaction which is witnessed by a close relative—which in any event will rarely be readily identifiable and observable) “are best left to be addressed in a case where they actually arise on the facts” (*Paul*, [123]). It is clear enough, though, that the majority was of the view that a claimant must be present at the scene of a medical setting *accident* or its immediate aftermath, and it is the accident that is the pivotal event, not any consequence thereof, no matter how horrifying or shocking.

The majority’s desire to limit this exception to the general principle was palpable in the judgment: “Unless the exception defined by the *Alcock* line of authority is to become the general rule […], a line must be drawn somewhere to keep the liability of negligent actors from such secondary harm within reasonable bounds” (*Paul*, [141]). The line the majority drew was between secondary victims who are present at the scene of an accident, have witnessed it, and have a close tie of love and affection with the victim—and then everyone else. The majority framed these as “restrictions which are reasonably straightforward, certain and comprehensible to the ordinary person” (*Paul*, [141]). One may doubt the force of that claim. The latter two restrictions are not entirely straightforward; for instance, what does it mean “to witness” an event, and what might constitute a “close tie of love and affection with the victim”? As for the former restriction of there being an accident, this raises even more concern about arbitrariness. As Lord Burrows noted in his dissent, in the context of clinical negligence, there will rarely be an accident (meaning an event external to the primary victim) and this needlessly denies recovery in almost all such cases, and what one means by “accident” is far from clear: it is just as possible to define an accident as an event external to the secondary victim as it is an event external to the primary victim. One may thus query whether the line drawn by the majority is too severe.

I am inclined, therefore, to tack closer to the opinion of Lord Burrows, treating the relevant event as the death (or serious illness) of the primary victim, thereby obviating the distinction between an accident and medical crisis. This approach is more persuasive in my view, as in these cases, it was witnessing the death or its immediate aftermath that caused the psychiatric illness to the secondary victims, and because it was reasonably foreseeable that they (as a person of reasonable fortitude) would suffer psychiatric illness as a consequence of the death. Provided foreseeability and the proximity/control factors are satisfied, as it was here, the secondary victim ought to be able to recover damages. Moreover, as Lord Burrows opined, it is irrelevant that there may be a significant time lag between the negligence and the death (or serious illness) of the primary victim: the temporal propinquity required is between the psychiatric illness and the *event caused* by the defendant’s negligence, not the negligence itself. And, as Lord Burrows also opined, it is a flawed objection that there may be a significant time between the accrual of the primary victim’s cause of action and the death (or serious illness) of the primary victim (and hence the suffering of the psychiatric illness by the secondary victim); the primary victim may not have any cause of action against the negligent defendant, or even where they do have a cause of action, the injury caused may be latent.

Ultimately, while much-needed clarity has been injected in this area of the law, it is difficult, reading the majority’s emphasis on the restricted scope of a medical practitioner’s duty, to envision a scenario in which a secondary victim could *ever* succeed in a clinical negligence context. Time will tell whether other jurisdictions will follow the reasoning of the majority or attribute more legal value to Lord Burrows’ rigorous and, might I add, justice-attuned dissent.

## Footnotes

1 The Supreme Court in fact heard three conjoined appeals from 16–18 May 2023 and the decision in *Paul* combines the three appeals as: (1) *Paul and another v Royal Wolverhampton NHS Trust*; (2) *Polmear and another v Royal Cornwall Hospitals NHS Trust*; and (3) *Purchase v Ahmed*. In each case the defendant applied to strike out the claim on the ground that as a matter of law it could not succeed. In this article, unless otherwise stated, I refer to *Paul* as the U.K. Supreme Court judgment on the three combined appeals.

2 The facts of the three cases were assumed to be true because they were strike out claims.

3 See e.g. *Baker v Bolton* (1808) 1 Camp 493; *Admiralty Comrs v SS Amerika* [1917] AC 38.

4 The Hillsborough disaster was a fatal human crush at a football (soccer) match at Hillsborough Stadium in Sheffield, England on April 15, 1989.

5
*Taylor v Somerset Health Authority* [1993] PIQR P262; *Sion v Hampstead Health Authority* [1994] 5 Med LR 170; *North Glamorgan NHS Trust v Walters* [2002] EWCA Civ 1792; *Shorter v Surrey and Sussex Healthcare NHS Trust* [2015] EWHC 614 (QB); *Liverpool Women’s Hospital NHS Foundation Trust v Ronayne* [2015] EWCA Civ 588. Many lawyers had long lamented the lack of clarity in the law regarding secondary victim claims in the clinical negligence context.

6 This means that mental distress (such as upset, grief, or anxiety) would not be recoverable.
